# Crimean–Congo Hemorrhagic Fever Virus Past Infections Are Associated with Two Innate Immune Response Candidate Genes in Dromedaries

**DOI:** 10.3390/cells11010008

**Published:** 2021-12-21

**Authors:** Sara Lado, Jan Futas, Martin Plasil, Tom Loney, Pia Weidinger, Jeremy V. Camp, Jolanta Kolodziejek, Dafalla O. Kannan, Petr Horin, Norbert Nowotny, Pamela A. Burger

**Affiliations:** 1 Research Institute of Wildlife Ecology, Department of Interdisciplinary Life Sciences, University of Veterinary Medicine Vienna, 1160 Vienna, Austria; sara.lado@meduniwien.ac.at; 2 Division of Infectious Diseases and Tropical Medicine, Department of Medicine I, Medical University of Vienna, 1090 Vienna, Austria; 3 Department of Animal Genetics, University of Veterinary Sciences Brno, 61242 Brno, Czech Republic; jfutas@vfu.cz (J.F.); plasilma@vfu.cz (M.P.); horin@ics.muni.cz (P.H.); 4 RG Animal Immunogenomics, CEITEC VETUNI Brno, 61242 Brno, Czech Republic; 5 College of Medicine, Mohammed Bin Rashid University of Medicine and Health Sciences, Dubai 505055, United Arab Emirates; tom.loney@mbru.ac.ae (T.L.); Norbert.Nowotny@vetmeduni.ac.at (N.N.); 6 Viral Zoonoses, Emerging and Vector-Borne Infections Group, Institute of Virology, University of Veterinary Medicine Vienna, 1210 Vienna, Austria; Pia.Weidinger@vetmeduni.ac.at (P.W.); Jeremy.Camp@meduniwien.ac.at (J.V.C.); Jolanta.Kolodziejek@vetmeduni.ac.at (J.K.); 7 Center for Virology, Medical University of Vienna, 1090 Vienna, Austria; 8 Al Ain City Municipality, Al Ain 505055, United Arab Emirates; Dafalla.Ahmed@aam.gov.ae

**Keywords:** zoonosis, vector-borne infection, tick, in-solution hybridization capture, Old World camel, Camelus dromedarius

## Abstract

Dromedaries are an important livestock, used as beasts of burden and for meat and milk production. However, they can act as an intermediate source or vector for transmitting zoonotic viruses to humans, such as the Middle East respiratory syndrome coronavirus (MERS-CoV) or Crimean–Congo hemorrhagic fever virus (CCHFV). After several outbreaks of CCHFV in the Arabian Peninsula, recent studies have demonstrated that CCHFV is endemic in dromedaries and camel ticks in the United Arab Emirates (UAE). There is no apparent disease in dromedaries after the bite of infected ticks; in contrast, fever, myalgia, lymphadenopathy, and petechial hemorrhaging are common symptoms in humans, with a case fatality ratio of up to 40%. We used the in-solution hybridization capture of 100 annotated immune genes to genotype 121 dromedaries from the UAE tested for seropositivity to CCHFV. Through univariate linear regression analysis, we identified two candidate genes belonging to the innate immune system: *FCAR* and *CLEC2B.* These genes have important functions in the host defense against viral infections and in stimulating natural killer cells, respectively. This study opens doors for future research into immune defense mechanisms in an enzootic host against an important zoonotic disease.

## 1. Introduction

Old and New World camelids (family Camelidae) are recognized not only as multipurpose animals adapted to extreme environments—providing milk, meat, and wool—but also for their potential in combating infectious diseases. The immune system of camels shows unique features, such as a special type of antibodies (so-called nanobodies), somatic hypermutations in T-cell receptor genes, and low polymorphism of the major histocompatibility complex (MHC) genes. Camelids have the unique ability to generate homodimeric immunoglobulins (Igs) in addition to conventional antibodies, where the antigen-binding fragment of these specific IgGs is reduced to a single variable domain lacking the light chain [1]. Due to their reduced size and resistance to high temperatures and chemical compounds, these nanobodies can be used for both clinical applications and antiviral therapy by transporting therapeutic agents into different body parts, as well as crossing the blood–brain barrier [2]. Recent studies have shown that nanobodies produced by camelids can effectively neutralize betacoronaviruses [3,4] and block SARS-CoV-2 infection [5].

Human-induced climate change, population growth, declines in biodiversity, and land-use change have been major drivers of the evolution and spread of zoonotic diseases [6]. Camelids have been identified as reservoirs for several zoonotic agents (see [7]). Due to increased consumption and contact with camel products, camels represent a significant point source for zoonotic disease transmission to humans. Knowledge of camel-borne diseases, clinical signs, and pathways of transmission is thus important to mitigate the human risks of camel-associated zoonoses. The dromedary (*Camelus dromedarius*), one of the three Old World camel species, has been identified as a reservoir host and the primary source of human infections of Middle East respiratory syndrome coronavirus (MERS-CoV; [8]), with spillover to humans and other livestock such as sheep occurring from direct or indirect contact with infected camels [9]. Additionally, there is evidence that camels serve as the primary reservoir of Crimean–Congo hemorrhagic fever virus (CCHFV) in some endemic foci, with high seropositivity and virus detected in both camel sera and camel-associated ticks [10,11].

CCHFV (order Bunyavirales, family Nairoviridae, genus *Orthonairovirus*) is a tick-borne, geographically widespread virus found across Europe, Asia, Africa, the Middle East, and the Indian subcontinent [12]. The transmission cycle involves *Hyalomma* ticks as reservoirs, with livestock, such as cattle, goats, and (in some regions) camels, as the principal amplifying hosts [13]. The genomic diversity of CCHFV is surprisingly high [13], partly as a result of the animal trade between different regions that has allowed for the relatively frequent reassortment of viral gene segments to occur [13]. Spillover into humans typically occurs through tick bites, resulting in a severe (or even fatal) hemorrhagic fever disease. Transmission may also occur as a result of exposure to tissues from infected animals during and immediately after slaughter [10,14]. Human-to-human transmission of CCHFV can occur but is limited to nosocomial settings, requiring direct contact with fluids from infected patients [12]. There is no apparent disease in dromedaries after the bite of infected ticks; in contrast, fever, myalgia, dizziness, neck pain, stiffness, tachycardia, lymphadenopathy, and petechial hemorrhaging are common symptoms in humans, with a case fatality ratio of up to 40% [15].

Several outbreaks of CCHFV have occurred in the Arabian Peninsula, specifically in the United Arab Emirates (UAE) in 1979 [16] and in Oman during 1994–1995 [13], both linked to nosocomial transmission. However, there is evidence of direct spillover to humans in recent years, and current studies have clearly demonstrated that CCHFV is endemic in dromedaries and camel ticks (*Hyalomma dromedarii*) in the UAE [10,11,14]. Although information is available on CCHFV prevalence, epidemiology, and genetic diversity [10,13,14,15,17,18,19], little is known about the immune responses of camels to this zoonotic pathogen and its underlying genetic basis. After a cross-sectional survey of dromedary camels and ticks in the UAE to determine their exposure status [10], we tried to understand the underlying genetic diversity in seropositive dromedaries at three different areas within the UAE. Following up on our previous work with MERS-CoV [8], we used the same samples and data generated from a target enrichment approach, along with the in-solution hybridization capture of 100 annotated immune genes [20], to genotype a larger number of dromedaries tested for seropositivity to CCHFV. With our work, we open doors for future research, including large-scale screening for genes underlying defense mechanisms in enzootic hosts against an important zoonotic disease.

## 2. Materials and Methods

### 2.1. Sampling and CCHFV Characterization

In this study, we used serum samples collected from a total of 121 dromedaries within an ongoing public health surveillance program in the UAE approved by the Al Ain City Municipality, as described by Lado et al. [8] and Camp et al. [10]. Briefly, the material was collected during two field seasons (March/April 2019 and October 2019) in three locations in the UAE: (1) the largest national livestock market (April 2019, *n* = 37; October 2019, *n* = 39); (2) a desert wildlife reserve with camels primarily used for tourism (April 2019, *n* = 30); and (3) a Bedouin family-owned farm with camels primarily raised for racing and trading (March 2019, *n* = 15) (Table S1). All dromedaries (aged ≥ 6 months) in the UAE have a subcutaneous identity microchip that is linked to a national database containing information on the camel’s age, sex, and geographic origin within the UAE. All camels were scanned for these microchips, and their demographic data were extracted from the national database.

Serum samples of all dromedaries were collected and stored at −80 °C at the College of Medicine, Mohammed Bin Rashid University of Medicine and Health Sciences, Dubai, UAE before shipment on dry ice to the University of Veterinary Medicine Vienna, Austria. All serum samples were screened for CCHFV-specific RNA and CCHFV-reactive antibodies, as described previously [10]. RNA was extracted from camel sera with a commercial kit (QIAamp Viral RNA Mini Kit, Qiagen) and tested for the presence of CCHFV nucleic acid using a commercial reverse transcription quantitative PCR (RT-qPCR) kit (RealStar^®^ CCHFV RT-PCR Kit, Altona). To determine the presence of CCHFV-reactive antibodies against the nucleoprotein of CCHFV, serum samples were tested using a commercial ELISA kit (ID Screen^®^ CCHF Double Antigen Multi-species, IDvet).

### 2.2. Probe Design and In-Solution Hybridization Capture Target Enrichment

Genomic analyses were performed with the same nucleic acid extracts described by Lado et al. [8] from all 121 dromedary serum samples, which were sent to Daicel Arbor Biosciences (Ann Arbor, MI, USA) for library construction, hybridization capture, and sequencing. Briefly, we used a target enrichment approach based on in-solution hybridization with biotinylated RNA probes and selected 100 immune response (IR) genes [8] from the most up-to-date dromedary (CamDro3) annotations (https://doi.org/10.5061/dryad.qv9s4mwb3 (accessed on 1 February 2021); [21]) for myBaits^®^ design. The selected regions were provided to Daicel Arbor Biosciences for bait design, and samples were sequenced on the Illumina NovaSeq 6000 platform on partial S4 flow cell lanes with 150-bp paired-end sequencing.

### 2.3. Variant Calling and Read-Based Imputation in IR Genes

In this study, we made use of the imputed dataset published by Lado et al. [8] (https://doi.org/10.5061/dryad.x69p8czh6, accessed on 28 June 2021). Briefly, after sequencing, adapter and quality trimming was performed, and then we merged VCF files for each individual into a single VCF file and only kept the SNPs that occurred in the target region where the 120-bp baits mapped. Thereafter, we performed the read-based imputation of SNPs to increase the number of variants to a final imputed dataset containing 3958 SNPs for all 121 dromedary samples.

### 2.4. Data Filtering

The quality control of the data, with ≤10% missingness after read-based imputation, was performed with PLINK 1.9 [22]. Relatedness was taken into consideration by detecting samples with unexpectedly high values of identity by state (IBS; i.e., >0.90) calculated in PLINK with the flags “--cluster” and “--matrix” to obtain the IBS similarity matrix. No pair of individuals showed identity by state higher than 0.88. Due to the low number of actively infected individuals, we continued the phenotype–genotype association analysis using univariate logistic regression including 114 dromedaries with antibody status tested by ELISA after removing seven samples: three samples positive for active virus infection, one sample ambiguous for antibody prevalence, and three samples missing age information. The dromedaries were split into two groups showing a prevalence (cases; *n* = 83) or absence (controls; *n* = 31) of CCHFV antibodies, indicating past infection, including 54 males, 57 females, and three with unknown sex. For the genotype data, we applied additional filtering steps to further reduce the possibility of capturing false positive variants and removed 1002 SNPs with low minor allele frequencies of 1% or less (MAF ≤ 1%) using the flag “--maf”. Furthermore, we filtered 27 SNPs out of Hardy–Weinberg equilibrium (“--hwe”; *p*-value = 0.0056428) corrected for a false discovery rate (FDR) based on the number of SNPs [23]. The final dataset consisted of 2929 SNPs genotyped in 114 dromedaries, including 54 males, 57 females, and three with unknown sex, which were grouped into 83 cases and 31 controls.

### 2.5. Univariate Logistic Regression Analysis for Phenotype–Genotype Association

The association of SNPs (passing filtering criteria stated above) with the phenotype CCHFV-positive (case) and -negative (control) for antibodies was tested by univariate logistic regression analysis accounting for sex, age, and population structure. First, we included the most informative PCs (1–3) as covariates using PLINK 1.9 with the flag “--pca”. After, we used PLINK 1.9 to perform the univariate logistic regression analysis by using “--logistic”, “--covar”, and “--adjust”. Genomic inflation factor λ (lambda) was calculated in PLINK after applying logistic regressed *p*-values, and for values lower than 1, we calculated lambda using R (R core team; [24]). Graphical representations of Manhattan and quantile–quantile (QQ) plots were obtained with the R packages qqman v.0.1.4 [25] and ggbio v.1.36.0 [26]. We identified significant SNPs on a cut-off of *p* ≤ 0.05 corrected for FDR [23]. Further SNPs located in genes with potential association with past CCHFV infection were ranked by the lowest uncorrected significant *p*-values [27]. Gene names were set based on functional annotations from Lado et al. ([20]; https://doi.org/10.5061/dryad.qv9s4mwb3, accessed on 28 June 2021), which we cross-referenced against GeneCards (https://www.genecards.org/, accessed on 7 July 2021). We used PLINK 1.9 to estimate allele frequencies and genotype counts, as well as to assess significant differences between positives and negatives, by using Fisher’s exact test with “--fisher”, “--model”, and “--GENO” for allele frequencies and genotype counts. Finally, as possible indicator of functional effects on splicing, we calculated the distance of significant SNPs to potential splice sites identified with NetGene2 v.2.42 (https://services.healthtech.dtu.dk/service.php?NetGene2-2.42, accessed on 16 November 2021; [28,29]). We conducted the analysis on both the reference and alternative (including minor alleles) sequence, but with no change in the results.

### 2.6. Linkage Disequilibrium (LD)-Based Gene-Set Test

We also performed an LD-based gene-set association analysis with PLINK 1.9, using the SNPs in each of the 100 IR genes as a separate set (Table S2). The empirical *p*-values were corrected for the multiple SNPs within a set (considering the LD between these SNPs). For this analysis, we applied the default values of the standard r-squared (--set-r2) = 0.5, *p*-value (--set-p) = 0.05, max number of SNPs (--set-max) = 5, and 10,000 permutations, representing a moderate setting of values.

## 3. Results

### 3.1. CCHFV Shedding and Antibody Prevalence in Dromedaries from the UAE

We detected 85 seropositive cases (70%), 35 seronegative cases (29%), and one borderline-positive case (1%) for CCHFV-specific antibodies by ELISA within the 121 dromedary samples, showing that most of these animals had experienced a CCHFV infection in the past. Animals with antibodies were present in all three locations in the UAE, showing equal exposure to past infections. Viral nucleic acids were only detected by RT-qPCR in three serum samples from the livestock market, showing a low number of active infections in dromedaries at the time of testing.

### 3.2. Phenotype–Genotype Association in Seropositive and Seronegative Dromedaries

**Figure 1:**
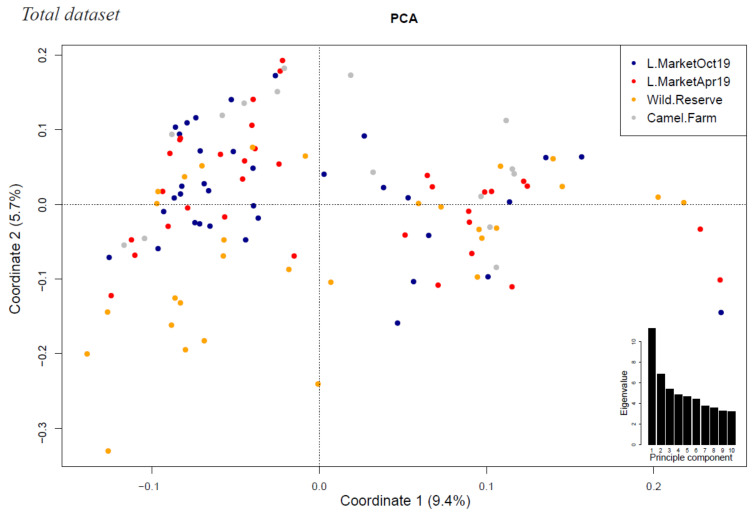
Principal component analysis of the population structure at three collection sites over two sampling periods. Variance explained by Coordinates 1 and 2 are given as percentages on the axes’ labels. Individual animals are plotted on the first two principal components, colored by sampling site (livestock market (“L.Market”) over two sampling periods (April and October 2019 denoted by red and dark blue, respectively), Dubai Desert Conservation Reserve (“Wild.Reserve”) denoted by yellow, and a Bedouin camel farm (“Camel.Farm”) denoted by grey). The inset shows a bar plot of the eigenvalues for the first 10 principal components.

**Figure 2:**
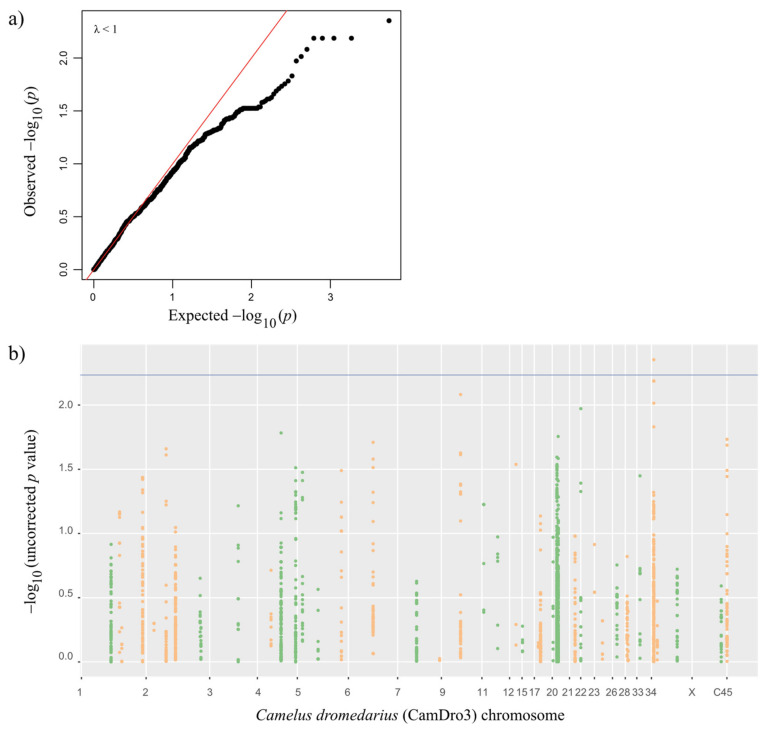
Univariate logistic regression results. (**a**) QQ plot and (**b**) Manhattan plot, with false discovery rate (FDR) threshold depicted in blue. −log_10_(*p*) values for SNPs alternate from green to orange to delineate chromosomes adjacent in the plots. C45 corresponds to Contig45, an unplaced scaffold in the CamDro3 reference.

As our samples originated from three different locations, we corrected for population structure to avoid population stratification bias and possible false positive associations. The genetic variation in the population explained by the first three most informative principal components (PCs 1–3) summed up to 19.7%, and we included these as covariates in addition to sex and age (Figure 1). We performed a univariate logistic regression with the complete dataset of 2929 SNPs imputed over 100 IR genes from 114 dromedaries, including 83 showing past infection (cases) and 31 with no detectable CCHFV-reactive antibodies (controls). The genomic inflation estimation lambda (based on median chi square) was lower than 1 (λ = 0.97). The quantile–quantile (QQ) plot (Figure 2a) with PCA correction showed that observed values generally followed the expected values, with an end tail characteristic of SNPs in potential association with the tested phenotypes.

The selection of an appropriate statistical significance threshold in phenotype–genotype association studies is critical to differentiate true positives from false positives and false negatives. Therefore, we decided to present significant markers that were selected based on a cut-off of *p* ≤ 0.05 corrected for FDR. In addition, we present the most significant SNPs ranked by the lowest uncorrected *p*-values (Table 1; Figure S1). We detected seven candidate SNPs (uncorrected *p* < 0.01), of which only one SNP was significant after using the FDR-corrected *p* < 0.00584, as displayed in the Manhattan plot (Figure 2b and Table 1). Nevertheless, due to an equivocal annotation in the CamDro3 reference genome, it was not clear whether two SNPS (chromosome (chr) 34:15810691,15809590) are located either in *KLRF2* (Killer cell lectin-like receptor subfamily F member 2) or *CLEC2B* (C-type lectin domain family 2 member B), as these two candidate genes were overlapping on this annotation. After the manual correction of the annotation based on the mRNA model of *Bos taurus KLRF2* (XM_015471290.2), we concluded that these SNPs are intronic of the *CLEC2B* gene. Thus, the seven top candidate SNPs (*p* < 0.01) were located within two genes on chromosomes 9 and 34: *FCAR* (Immunoglobulin Alpha Fc Receptor), which contained one intronic SNP (chr9:73819886), and *CLEC2B,* which contained six intronic SNPs, (Table 1). The closest distance of a significant SNP to a highly confident (H) splice site was 933 bp downstream in the *FCAR* gene (chr9:73820819) and 606 bp upstream in *CLEC2B* (chr34:15808984) (Table S3 and Figure S2).

We repeated the univariate logistic regression analysis with the initially called 760 SNPs after filtering for 25% of genotyping missingness (without imputation). Due to higher genotype missingness, we accepted slightly higher IBS values (i.e., 0.91) to account for relatedness, but we required similar criteria for HWE (0.00693) and MAF (<1%) thresholds as before and considered the population structure (first four PCs explaining 35% of the total variation). After filtering out 55 variants based on the Hardy–Weinberg exact test and 13 variants by the MAF threshold, 692 variants and 114 samples (83 cases and 31 controls) were included. With a lambda equal to 1 and the application of the FDR threshold (FDR = 0.0070), three SNPs from Granzyme B (*GZMB*) and one from *FCAR* were found to be significant (Figure S3). This significant *FCAR* SNP (chr9:73819886) was found to be the same SNP as one of the top 7 most significant SNPs in the main analysis. Among the potentially associated variants with slightly higher *p*-values, right below the FDR threshold, we identified two other SNPs (chr9: 73820233,73819171) from *FCAR* (*p*-values = 0.01286 and 0.01787, respectively). These two SNPs were found to be present within the top 20 most significant SNPs with the imputed dataset (*p*-value = 0.0236 and 0.0243, respectively), which we thus also considered to be a strong candidate. The three *GZMB* SNPs were located in an intergenic region, most likely as part of a repetitive sequence. No SNP from *CLEC2B* was among the top 20 most significant SNPs from the non-imputed dataset (Figure S3).

After calculating allele frequencies for the top seven candidate SNPs plus the two *FCAR* SNPs that were common to the top 20 most significant SNPs in both analyses, we detected significantly higher frequencies of the minor allele in the three *FCAR* sequences (chr9: 73819886, 73820233, and 73819171; *p* = 0.0011, 0.0030, and 0.0063, respectively) in CCHFV antibody-positive dromedaries (Table 1). In addition, the homozygote genotype counts for the minor allele in the same three *FCAR* SNPs were significantly higher (*p* = 0.0004, 0.0021, and 0.0073, respectively) in CCHFV antibody-positive camels than in negative ones (Table 1).

### 3.3. Linkage Disequilibrium (LD)-Based Gene-Set Test

To further test the robustness of our results, we applied a complementary approach by means of a gene-set association test using the complete dataset (114 individuals and 2929 SNPs). Of the 100 targeted IR genes, 40 were shown to have significant SNPs (uncorrected *p* < 0.05), including the gene *FCAR*, which was nominally significant (*p* = 0.0031). *FCAR* harbored 12 SNPs, of which 9 were significant. However, only one SNP in *FCAR* (Chr9:73819886) passed the independent significance r-squared-based threshold of 0.5. Though this gene showed a stronger signal for potential genotype–phenotype association, *CLEC2B* was not significant (Table S4).

## 4. Discussion

Diversity in the immune system coding part of the genome is an important indicator of an individual’s capacity to adapt to changing environments with different pathogens. The visible impacts of climate change on camels include the expansion of the geographical distribution of the species, more contact between animals and humans, and the consequently increased risk of emerging zoonotic diseases [30,31]. Different studies have demonstrated a high seroprevalence in camel populations regarding a variety of zoonotic pathogens with examples of camel-to-human transmission (reviewed in [7]). Thus, it is important to explore how particular pathogens affect immune genetic diversity in camels and other reservoirs, as well as how genetic variation influences adaptation to emerging zoonoses, habitat fragmentation, and climate change [32]. In comparison to other species, camels show higher resistance to some infectious diseases and environmental stress [33]. The results of our analysis of a set of 100 IR genes encoding parts of the innate and the adaptive immune system contribute to the understanding of genetic mechanisms underlying the defense against CCHFV infection.

### 4.1. Antibody Response against CCHFV and Seroconversion in Dromedaries

In this study, we used a set of samples from a previous study [10], taken from dromedaries at several locations in the UAE, in which a relatively high seroprevalence with confirmation of active CCHFV infections in both dromedaries and camel-associated ticks was determined. A recent review ([34]) nicely stated that all large mammalian hosts studied thus far (mostly domestic species) produce robust humoral immune responses capable of neutralizing virus in vitro. Importantly, these species are also typically asymptomatic—but viremic—following infection. Few experimental studies—and even fewer studies involving “closed” or tick-protected herds—have investigated the duration of the immune response in these hosts (past ~40 day post-infection). To our knowledge, experimental infection studies involving CCHFV in camelids have never been performed. The duration of the humoral response to CCHFV is therefore unknown, and whether re-infections occur is largely undetermined. Given the relatively high tick burden in the study population, we suspect that repeated exposure occurs, particularly in livestock markets when animals are concentrated and tick control measures are not robust. Though we cannot confirm that all sampled animals have been exposed, we suspect that it is highly likely due to similar serosurvey reports in other countries showing high ratios of seroconversion that increases with age but never reaches 100% in a study population [10,35,36,37,38]. Similarly, in our cohort, we noted an age-associated increase in seroprevalence, but the highest ratio of seropositivity never reached 100% despite a relatively high tick burden on all animals.

In general, possible explanations of seronegativity include that individuals might be (i) resistant to infection and therefore quickly recover without eliciting a detectable antibody response; (ii) resistant but use an immune mechanism other than antibody production to get protected; or (iii) susceptible, since they are non-responders to the virus (for any possible reason) and as such will not be protected upon reinfection. We do not think that a transient production of antibodies, another possible explanation, would be the major cause of the differences observed in our study. Seropositivity and seronegativity are clearly distinct phenotypes related to infection observed in camels under a comparable pathogen pressure. Although our data do not allow us to estimate the relative importance of all possible explanations, it is a matter of fact that there is a genetic difference observed between the two phenotypic groups for the two genes *CLEC2B* and *FCAR*. If we dare to speculate about the reasons for the observed differences, we would think that at least the majority of our seronegative camels were free of antibodies due to their increased resistance to infection based on either the quick elimination of the virus followed by the rather poor stimulation of antibody production or based on another mechanism of protection. Non-responding camels have probably been eliminated by natural selection, leaving only two kinds of animals capable of surviving in the population under infectious pressure. The quick responders remain seronegative and/or need some time to respond. For them, a longer exposure to viral antigens leads to the stimulation of antibody production and protection. This would also explain the lack of clinical disease in the population.

### 4.2. Candidate IR Genes Associated with a Past CCHFV Infection in Dromedaries

Adaptive immunity is a highly specific immune response and its variability is subject to different selective pressures, but innate immunity is an efficient but less specific first protection against many pathogens [39]. In this study, we identified two main candidate genes potentially associated with a past CCHFV infection: *CLEC2B* (chr 34), coding for a C-type lectin, and *FCAR* (chr 9), encoding a receptor for the Fc fragment of IgA. Both candidate genes have relevant functions within the immune system. None of the significant SNPs were in close vicinity (<500 bp) to splice sites indicating possible functional effects on splicing.

*CLEC2B*: Only one SNP (chr39:15810691) was significant in the main genotype-phenotype association test after FDR correction, although five other SNPs had *p*-values ≤ 0.01 (Table 1). Additionally known as activation-induced C-type lectin (*AICL*), *CLEC2B* belongs to the CLEC2 family, which comprises ligands for natural killer (NK) gene complex-encoded C-type lectin-like receptors [40]. In humans, the encoded type 2 transmembrane protein may function as an activation-induced antigen with roles in inflammation and immune response [41]. *CLEC2B* was reported as an important gene in NK cells stimulating NK cell effector functions, such as cytotoxicity and cytokine secretion [42]. NK cells are lymphocytes that are part of the innate immune system and are critical in the defense against virus-infected cells, intracellular bacteria, parasites, fungi, and malignant cells [43]. On the other hand, we cannot exclude the possibility that *CLEC2B* SNPs represent positional markers for a huge family of CLEC genes present on chromosome 34. Most research has focused on the ability of C-type lectins to function in innate and adaptive immune responses, although they have increasingly been recognized to have other important roles as well (see [44]). C-type lectin receptors on myeloid cells, for instance, monitor their environment and sense pathogen-associated molecular patterns and cell-associated C-type lectins to subsequently modulate the activity of immune cells [45].

*FCAR*: This gene is a member of the immunoglobulin gene superfamily and encodes a receptor for the Fc region of IgA. Fc receptors play a central role in maintaining the homeostatic balance in the immune system. In humans, several SNPs have been identified through Fc receptor sequence analysis with known functional relevance and disease association, including host-defense mechanisms against viral infections [46]. The *FCAR* receptor is a transmembrane glycoprotein present on the surface of myeloid lineage cells, where it mediates immunologic responses to pathogens, triggering several immunologic defense processes including phagocytosis, antibody-dependent cell-mediated cytotoxicity, and the stimulation of the release of inflammatory mediators [47]. In our study, a significantly higher (*p* < 0.01) frequency was observed for the minor allele of the three *FCAR* SNPs in CCHFV seropositive dromedaries (Table 1). This observation of higher MAF within *FCAR* might suggest that these dromedaries are more susceptible to CCHFV infection. Accordingly, the homozygote alternative (minor) genotype of *FCAR* was significantly more frequent in our case group (*p* ≤ 0.005). The odds ratio was also higher than three for this allele, which might indicate that the odds of exposure among past CCHFV infection cases are greater than the odds of exposure among controls, showing a risk factor for the infection. This needs to be corroborated by additional sequencing and haplotype analyses, including a more large-scale sampling approach. It is also possible that *FCAR* represents a positional marker in linkage with some other immunoglobulin-like (LILR) genes (haplotypes) of the leukocyte receptor complex. These genes were not represented in our panel of 100 IR genes due to their high sequence homology and complexity, which had not been resolved at the time of panel design.

In summary, we identified two important candidate genes related to the innate immune system in dromedaries from the UAE. The functional importance of these genes in response to CCHFV infections in dromedaries needs to be investigated in more controlled in vitro and in vivo experiments.

### 4.3. Challenges and Future Steps

The nonrandom distribution of SNPs observed between previously infected and non-infected individuals indicates that they are genetically different, which consequently demands further investigation, especially in terms of their immune mechanisms. Future case–case control studies need to include dromedary populations outside of the UAE. The next important step will be to investigate the expression and functional pathways of the identified candidate IR genes responsible for higher resistance to CCHFV infection. However, it is not always realistic to comply with the requirements of functional analyses for our type of study and the confirmation of the statistical data presented in the manuscript. For a functional follow-up study, we must obtain access to samples suitable for RNA extraction that are collected in a specific situation, i.e., at a time and in a cell population when/where differences in gene expression can be expected. Likewise, if we interpret seroconversion in terms of the capacity of the immune system to react/non-react during infection, we would need a to analyze gene expression at the time of infection, which would require experimental infection in vitro and/or in vivo. This is a different situation compared to the analyses of sick and healthy animals where changes in gene expression are expected to occur. We could check whether camels with and without antibodies differ in the expression of selected genes at any moment, but the information value and interpretation of results would be quite uncertain and not directly related to a confirmation of statistical data. Another issue is the identification and isolation of cell populations suitable/informative for gene expression analysis. There is no information on the tissue expression of the genes associated with the phenotypes analyzed in camels, and interspecific extrapolation cannot be straightforward, especially for the *CLEC2B* gene. Moreover, the isolation of specific cell types in camels is not a routine procedure due to their insufficient characterization in this species and consequent lack of standardized tools (reagents). Lastly, for specific functional studies, we would need activated samples; however, CCHFV is a biosafety level (BSL) class 3 (to 4) agent, which needs to be handled at least in a BSL-3 laboratory. Such a laboratory is not available in the UAE, and in order to send the samples to Austria, where they were analyzed for this study, they had to be inactivated.

Camels (and some other livestock) are competent amplifying hosts of CCHFV and therefore present a risk of infection for humans who work closely with livestock—particularly those in the livestock trade or abattoir workers. Preventing human infections is especially important considering the high case fatality ratio of up to 40%. Controlling ticks on livestock is one recommended method for reducing CCHFV transmission activity and the enzootic maintenance of the virus, but acaricides are not often used [10,14]. As no commercial veterinary CCHFV vaccine is available for livestock, it is important to understand the immune response of CCHFV-amplifying hosts, such as camels, to assist in vaccine design. Currently, there is also no vaccine against human infection with CCHFV, and available post-exposure prophylaxis is non-specific (e.g., ribavirin). Therefore, understanding the course of infection in camels, specifically, may benefit the management of human disease in endemic regions via the development of nanobodies as specific therapeutics.

## Figures and Tables

**Table 1:** Significant SNPs (*p* < 0.01) located in candidate immune response genes, allele frequencies of the minor alleles, and genotype counts (homozygote minor/heterozygote/homozygote major allele) in CCHFV-positive (cases) and -negative (controls) camels.

Chr	Position (Minor/Major Allele)	Gene	*p*-Value	Allele Freq. Minor Allele				Genotypes		
				Cases	Controls	Exact *p*-Value	Odds Ratio	Cases	Controls	Exact *p*-Value	
34	A15810691G	*CLEC2B*	0.0044 **	0.2222	0.2759	0.4716	0.7500	4/28/49	2/12/15	0.6212	
34	G15812682A	*CLEC2B*	0.0065 *	0.2289	0.2903	0.3878	0.7257	4/30/49	2/14/15	0.5572	
34	T15812711C	*CLEC2B*	0.0065 *	0.2289	0.2903	0.3878	0.7257	4/30/49	2/14/15	0.5572	
34	G15818303A	*CLEC2B*	0.0065 *	0.2289	0.2903	0.3878	0.7257	4/30/49	2/14/15	0.5572	
34	C15818368T	*CLEC2B*	0.0065 *	0.2289	0.2903	0.3878	0.7257	4/30/49	2/14/15	0.5572	
9	G73819886A	*FCAR*	0.0083 *	0.4329	0.1935	0.0011 *	3.1810	11/49/22	2/8/21	0.0004 *	
34	A15809590C	*CLEC2B*	0.0097 *	0.2289	0.2833	0.4827	0.7509	4/30/49	2/13/15	0.6277	
9	C73820233A	*FCAR*	0.0236	0.4036	0.1935	0.0030 *	2.8200	10/47/26	2/8/21	0.0021 *	
9	G73819171A	*FCAR*	0.0243	0.3735	0.1774	0.0063 *	2.7640	10/42/31	2/7/22	0.0073 *	

* significant *p* < 0.01; ** significant after FDR correction.

## Data Availability

Raw FASTQ files were deposited on the European Nucleotide Archive (ENA) [ERS5621787 (SAMEA7874536)–ERS5621907 (SAMEA7874656)]. VCF files, target region, and bait sequences are available on dryad together with the scripts file (https://doi.org/10.5061/dryad.x69p8czh6, accessed on 28 September 2021). Additional material requests can be addressed to Pamela.Burger@vetmeduni.ac.at.

## Supplementary Materials

The following are available online at https://www.mdpi.com/article/10.3390/cells11010008/s1, Figure S1: principal component analysis of the population structure at three collection sites over two sampling periods for the non-imputed dataset; Figure S2: Significant SNP positions in the intronic regions of *FCAR* and *CLEC2B*; Figure S3: Manhattan and QQ plot for the total dataset and non-imputed dataset; Table S1: Sample information and phenotype characterization; Table S2: List of selected IR genes; Table S3: Potential splice sites in the *FCAR* and *CLEC2B* gene; Table S4: Linkage Disequilibrium-based haplotype (gene-set) test.
