# Supplementary material for: Crimean–Congo Hemorrhagic Fever Virus Past Infections Are Associated with Two Innate Immune Response Candidate Genes in Dromedaries
Source: Cells. 2021 Dec 21;11(1):8. doi: 10.3390/cells11010008 (PMC8750074; doi:10.3390/cells11010008)
Supplement: Supplementary file 1 [file cells-11-00008-s001.zip › cells-1501230-supplementary.pdf]

Supplementary Materials for

Crimean-Congo hemorrhagic fever virus past infections are associated with two innate immune response candidate genes in dromedaries

S. Lado[1,2], J. Futas[3,4], Martin Plasil[3,4], T. Loney[5], P. Weidinger[6], J. V. Camp[6,7], J. Kolodziejek[6], D. O. Kannan[8], P. Horin[3,4], N. Nowotny[5,6] and P. A. Burger[1,*]

1 Research Institute of Wildlife Ecology, Department of Interdisciplinary Life Sciences, University of Veterinary Medicine Vienna, Vienna, Austria; sara.lado@meduniwien.ac.at (S.L.); pamela.burger@vetmeduni.ac.at (P.A.B.)
2 Division of Infectious Diseases and Tropical Medicine, Department of Medicine I, Medical University of Vienna, Vienna, Austria
3 Department of Animal Genetics, University of Veterinary Sciences Brno, Brno, Czech Republic; plasilma@vfu.cz (M.P.); jfutas@vfu.cz (J.F.); horin@ics.muni.cz (P.H.)
4 RG Animal Immunogenomics, CEITEC VETUNI Brno, Brno, Czech Republic
5 College of Medicine, Mohammed Bin Rashid University of Medicine and Health Sciences, Dubai, United Arab Emirates; tom.loney@mbru.ac.ae (T.L.); Norbert.Nowotny@vetmeduni.ac.at (N.N.)
6 Viral Zoonoses, Emerging and Vector-Borne Infections Group, Institute of Virology, University of Veterinary Medicine Vienna, Vienna, Austria; Pia.Weidinger@vetmeduni.ac.at (P.W.); Jeremy.Camp@meduniwien.ac.at (J.V.C.); Jolanta.Kolodziejek@vetmeduni.ac.at (J.K.)
7 Center for Virology, Medical University of Vienna, Vienna, Austria
8 Al Ain City Municipality, Al Ain, United Arab Emirates; Dafalla.Ahmed@aam.gov.ae (D.O.K.)

* Correspondence: pamela.burger@vetmeduni.ac.at, +431250777141

This PDF file includes:

Figures S1, S2 and S3
Tables S1, S2, S3 and S4

Non-imputed dataset

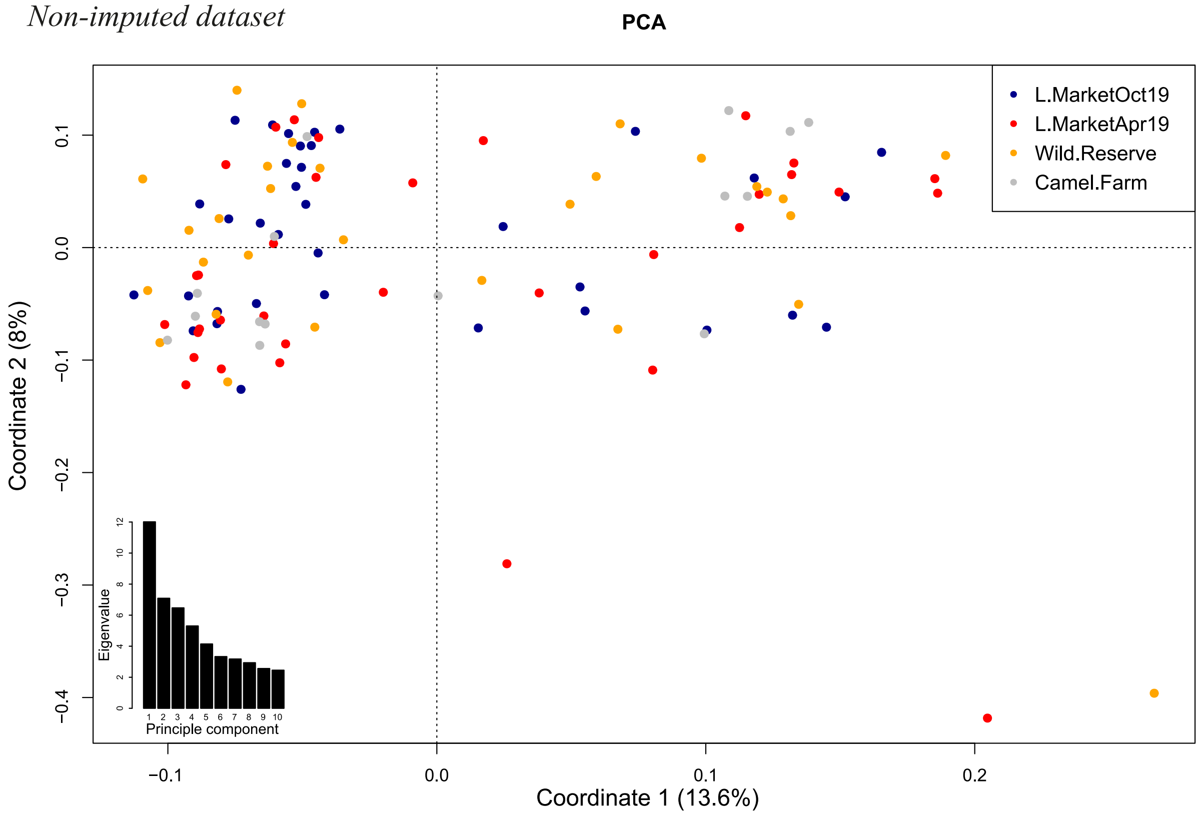

Figure S1. Principal Component Analysis of the population structure at three collection sites over two sampling periods for the non-imputed dataset. Variation explained by PC1 and PC2 are depicted in percentages. Individual animals are plotted on the first two principal components, coloured by sampling site (livestock market ["L.Market"], over two sampling periods (April and October 2019, dark and light blue, respectively); Dubai Desert Conservation Reserve ["Wild.Reserve"], dark red; and a Bedouin camel farm ["Camel.Farm"], pink). The inset shows a barplot of the eigenvalues for the first 10 principal components.

***FCAR* (chr9: 73807328 - 73825701)**

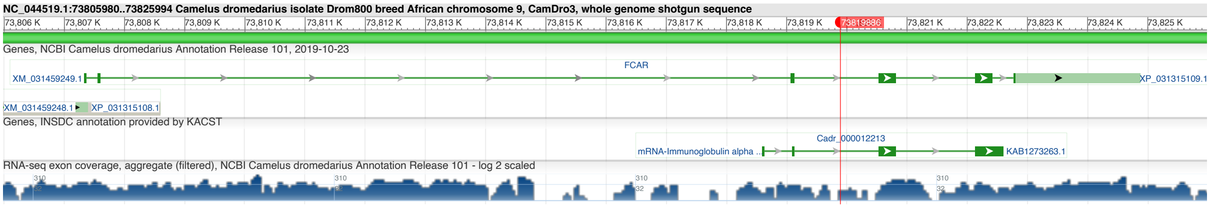

***CLEC2B* (chr34: 15807876 - 15823606)**

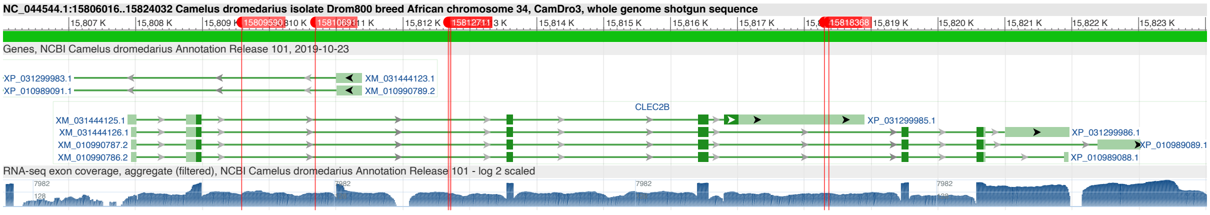

Figure S2. Significant SNP positions in the intronic regions of *FCAR* and *CLEC2B*. Significant SNPs (Table 1) are highlighted in red. The position numbering follows the dromedary reference genome CamDro3 (GCF_000803125.2).

a) Total dataset
(three first PC)

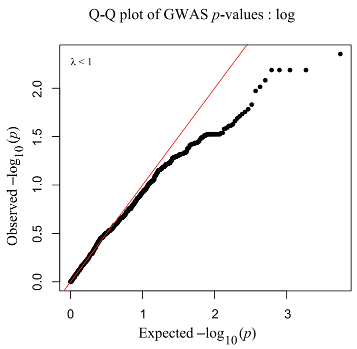

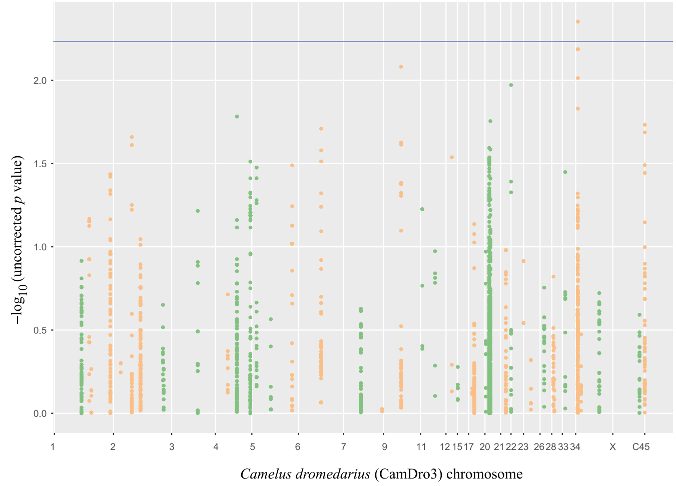

Chr	Position	Gene	*p* -value
34	15810691	*CLEC2B*	0.0044*
34	15812682	*CLEC2B*	0.0065
34	15812711	*CLEC2B*	0.0065
34	15818303	*CLEC2B*	0.0065
34	15818368	*CLEC2B*	0.0065
9	73819886	*FCAR*	0.0083
34	15809590	*CLEC2B*	0.0097
22	298936	*GZMM*	0.0107
34	15817796	*CLEC2B*	0.0148
5	8553067	*PTPN4*	0.0165
20	23101961	*HLA-A-24*	0.0176
Contig45	331563	*DDX58*	0.0185
6	95025252	*TRAF3*	0.0195
Contig45	338479	*DDX58*	0.0205
2	95084365	*TLR10*	0.0219
9	73820233	*FCAR*	0.0236
9	73819171	*FCAR*	0.0243
2	95081295	*TLR10*	0.0245
20	20684519	*HLA-DPA1*	0.0255
20	23101931	*HLA-A-24*	0.0261

b) Non-imputed dataset
(four first PC)

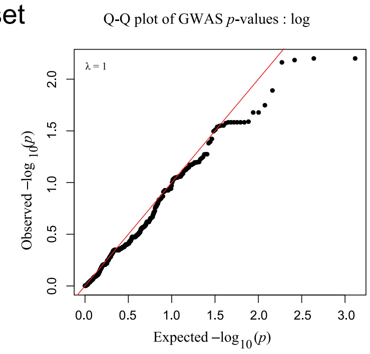

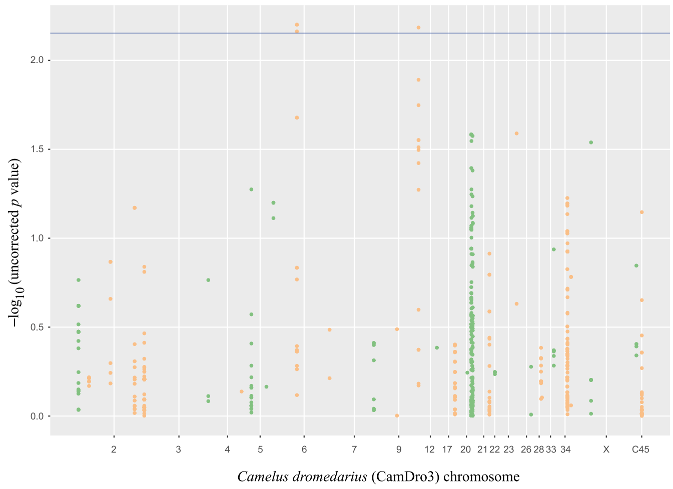

Chr	Position	Gene	*p* -value
6	34079760	*GZMB*	0.0063*
6	34079763	*GZMB*	0.0063*
9	73819886	*FCAR*	0.0065*
6	34079992	*GZMB*	0.0069*
9	73820233	*FCAR*	0.0129
9	73819171	*FCAR*	0.0179
6	34083198	*GZMB*	0.0210
6	34084005	*GZMB*	0.0210
23	30846690	*IL10*	0.0258
20	20831045	*HLA-DOB*	0.0261
20	20831074	*HLA-DOB*	0.0261
20	20832742	INTERGENIC	0.0261
20	20834325	INTERGENIC	0.0261
20	20834645	INTERGENIC	0.0261
20	20834956	INTERGENIC	0.0261
20	23102048	*HLA-A-24*	0.0266
9	73820723	*FCAR*	0.0281
9	73820724	*FCAR*	0.0281
20	21033478	*HLA-DRB1*	0.0284
37	27539160	INTERGENIC	0.0289

Figure S3. Manhattan and QQ plot for the total dataset and non-imputed dataset. Top 20 most significant SNPs are presented. FDR corrected thresholds are represented in blue in the Manhattan plot, and *p*-values with * are significant after FDR thresholds. a) Total dataset; b) non-imputed dataset. C45 corresponds to Contig45, an unplaced scaffold.

Supplementary tables

Table S1. Sample information and phenotype characterization for virus shedding (RT-qPCR) and antibody prevalence (ELISA) from blood sera.

Original code	Lab code	Sampling place	Sex (M/F)	Age (yr) approx.	Chip ID	CCHFV RT-qPCR JVC	CCGFV ELISA	Samples used in association tests
1	Drom1458	Livestock Market, UAE Oct19	M	2	900182001697895	neg	pos. 170.5	X
2	Drom1459	Livestock Market, UAE Oct19	M	4 m	NO CHIP INFO	neg	neg. 4.9	X
3	Drom1460	Livestock Market, UAE Oct19	M	2	900215000003532	neg	pos. 227.8	X
4	Drom1461	Livestock Market, UAE Oct19	-	-	900111881027297	neg	pos. 70.2	
5	Drom1462	Livestock Market, UAE Oct19	M	1	900057600126219	neg	pos. 143.0	X
6	Drom1463	Livestock Market, UAE Oct19	M	3 m	NO CHIP INFO	neg	pos. 192.4	X
7	Drom1464	Livestock Market, UAE Oct19	M	2 m	NO CHIP INFO	neg	weak 38.7	
8	Drom1465	Livestock Market, UAE Oct19	F	3	900057600122200	neg	pos. 153.5	X
9	Drom1466	Livestock Market, UAE Oct19	F	3	900215000005008	neg	pos. 118.5	X
10	Drom1467	Livestock Market, UAE Oct19	F	5	985007841277728	neg	pos. 222.5	X
11	Drom1468	Livestock Market, UAE Oct19	F	6	784010050031772	neg	pos. 228.9	X
13	Drom1470	Livestock Market, UAE Oct19	F	1	784010050046584	neg	pos. 184.6	X
14	Drom1471	Livestock Market, UAE Oct19	F	6	992001000331305	neg	pos. 230.1	X
23	Drom1480	Livestock Market, UAE Oct19	F	6	784010050267117	neg	pos. 198.1	X
25	Drom1482	Livestock Market, UAE Oct19	F	3	900057600121775	neg	pos. 236.2	X
27	Drom1484	Livestock Market, UAE Oct19	F	6 m	784010050465212	neg	pos. 195.9	X
28	Drom1485	Livestock Market, UAE Oct19	M	5 m	NO CHIP INFO	neg	pos. 176.7	X
29	Drom1486	Livestock Market, UAE Oct19	M	6 m	NO CHIP INFO	neg	pos. 180.3	X
31	Drom1488	Livestock Market, UAE Oct19	M	2	908182001493742	neg	pos. 217.7	X
32	Drom1489	Livestock Market, UAE Oct19	M	2	991001002574519	neg	pos. 205.1	X
35	Drom1492	Livestock Market, UAE Oct19	F	6	900057600126791	neg	pos. 204.1	X
37	Drom1494	Livestock Market, UAE Oct19	F	5	968000002916753	neg	pos. 216.0	X
38	Drom1495	Livestock Market, UAE Oct19	M	6	985007841229562	neg	pos. 257.7	X
39	Drom1496	Livestock Market, UAE Oct19	F	6	784010050348103	neg	pos. 147.4	X
42	Drom1499	Livestock Market, UAE Oct19	F	3	784019000006947	neg	neg. 12.8	X
43	Drom1500	Livestock Market, UAE Oct19	F	6	985007841219484	neg	pos. 216.5	X
45	Drom1502	Livestock Market, UAE Oct19	M	5	985007841209387	neg	pos. 170.2	X
47	Drom1504	Livestock Market, UAE Oct19	F	6	784010050073661	neg	pos. 174.2	X
48	Drom1505	Livestock Market, UAE Oct19	F	5	784019000006158	neg	pos. 186.8	X
51	Drom1508	Livestock Market, UAE Oct19	F	2	985007841357609	neg.	neg. 9.9	X
54	Drom1511	Livestock Market, UAE Oct19	M	2	900057600121585	neg	pos. 233.2	X

56	Drom1513	Livestock Market, UAE Oct19	F	2	784010050551133	pos. 37.3	neg. 8.0	
69	Drom1526	Livestock Market, UAE Oct19	F	6	784010050079111	neg	pos. 224.6	X
72	Drom1529	Livestock Market, UAE Oct19	-	-	991001001739927	neg	pos. 222.7	
79	Drom1536	Livestock Market, UAE Oct19	F	6	784010050079139	neg	pos. 186.2	X
85	Drom1542	Livestock Market, UAE Oct19	F	4	784010050140963	neg	pos. 217.4	X
87	Drom1544	Livestock Market, UAE Oct19	M	1	900057600121595	neg.	neg. 7.0	X
88	Drom1545	Livestock Market, UAE Oct19	-	6 m	NO CHIP INFO	neg.	neg. 28.5	X
90	Drom1547	Livestock Market, UAE Oct19	F	6	784010050294118	neg	pos. 223.2	X
NSB5	Drom1823	Livestock Market, UAE April19	M	2	900111881038114	neg.	neg. 3.1	X
NSB8	Drom1826	Livestock Market, UAE April19	M	2	900111881038106	neg.	neg. 3.3	X
NSB10	Drom1827	Livestock Market, UAE April19	F	2	784010050550508	neg.	pos. 153.9	X
NSB11	Drom1828	Livestock Market, UAE April19	M	2	784010050550691	neg.	pos. 189.3	X
NSB12	Drom1829	Livestock Market, UAE April19	-	-	NO CHIP INFO	neg.	neg. 3.3	
NSB13	Drom1830	Livestock Market, UAE April19	F	4	784010050423283	very weak pos.	neg. 3.5	
NSB14	Drom1831	Livestock Market, UAE April19	M	2	900111881038113	neg.	neg. 3.1	X
NSB16	Drom1833	Livestock Market, UAE April19	F	4-5	634078000075327	neg.	pos. 99.4 (1:10)	X
NSB17	Drom1834	Livestock Market, UAE April19	F	8	784010050077291	neg.	pos. 150.4	X
NSB18	Drom1835	Livestock Market, UAE April19	F	2	900182001414799	neg.	pos. 157.0	X
NSB19	Drom1836	Livestock Market, UAE April19	M	1	991001002575031	neg.	pos. 120.7	X
NSB20	Drom1837	Livestock Market, UAE April19	M	2	900111880935178	neg.	pos. 152.1	X
NSB21	Drom1838	Livestock Market, UAE April19	M	3	985007841400429	neg.	pos. 173.0	X
NSB23	Drom1840	Livestock Market, UAE April19	M	1	991001002570111	neg.	neg. 18.1	X
NSB30	Drom1847	Livestock Market, UAE April19	F	4	784010050229794	neg.	pos. 166.9	X
NSB31	Drom1848	Livestock Market, UAE April19	F	6	784010050243508	neg.	neg. 7.2	X
NSB33	Drom1850	Livestock Market, UAE April19	M	1-2	992001000330620	neg.	neg. 24.7	X
NSB34	Drom1851	Livestock Market, UAE April19	F	2	991001002575035	neg.	neg. 3.9	X
NSB35	Drom1852	Livestock Market, UAE April19	F	3	991001002574462	neg.	neg. 3.2	X
NSB36	Drom1853	Livestock Market, UAE April19	M	3	991001002575745	neg.	neg. 3.1	X
NSB37	Drom1854	Livestock Market, UAE April19	M	2-3	992001000330472	neg.	neg. 3.0	X
NSB38	Drom1855	Livestock Market, UAE April19	M	3	991001002575920	neg.	neg. 3.3	X
NSB39	Drom1856	Livestock Market, UAE April19	F	6	784010050365484	neg.	pos. 173.7 (1:10)	X
NSB40	Drom1857	Livestock Market, UAE April19	-	2 m	NO CHIP INFO	neg.	neg. 18.3	X
NSB41	Drom1858	Livestock Market, UAE April19	F	4	985007841359958	neg.	pos. 187.0	X
NSB42	Drom1859	Livestock Market, UAE April19	F	4	784010050067053	neg.	pos. 155.1	X
NSB43	Drom1860	Livestock Market, UAE April19	M	1-2	992001000330719	neg.	pos. 124.5	X
NSB44	Drom1861	Livestock Market, UAE April19	F	6	784010050516433	neg.	pos. 110.1	X
NSB45	Drom1862	Livestock Market, UAE April19	F	3	784010050028578	neg.	pos. 179.3	X
NSB46	Drom1863	Livestock Market, UAE April19	F	6	991001002575819	neg.	pos. 173.6	X
NSB47	Drom1864	Livestock Market, UAE April19	M	3	991001002575899	neg.	neg. 3.2	X
NSB48	Drom1865	Livestock Market, UAE April19	M	1-2	990001000053606	neg.	neg. 3.7	X
NSB49	Drom1866	Livestock Market, UAE April19	F	3	900111881038306	neg.	neg. 13.2	X

NSB50	Drom1867	Livestock Market, UAE April19	-	2 m	NO CHIP INFO	neg.	pos. 51.7	X
NSB51	Drom1868	Livestock Market, UAE April19	M	2	991001002574752	pos. 36.7	neg. 3.4	
NSB52	Drom1869	Livestock Market, UAE April19	M	1-2	900074001585559	neg.	neg. 24.9	X
NSB54	Drom1871	Livestock Market, UAE April19	F	5	784010050359111	neg.	pos. 162.9	X
NSw20	Drom1872	Wildlife Reserve, Al Maha	M	15	NO CHIP INFO	neg	pos. 173.0	X
NSw21	Drom1873	Wildlife Reserve, Al Maha	M	13	NO CHIP INFO	neg	pos. 167.0	X
NSw22	Drom1874	Wildlife Reserve, Al Maha	M	17	NO CHIP INFO	neg	neg. 22.4	X
NSw23	Drom1875	Wildlife Reserve, Al Maha	F	14	NO CHIP INFO	neg	pos. 170.0	X
NSw25	Drom1877	Wildlife Reserve, Al Maha	M	9	NO CHIP INFO	neg	pos. 190.4	X
NSw26	Drom1878	Wildlife Reserve, Al Maha	M	7	NO CHIP INFO	neg	pos. 144.6	X
NSw27	Drom1879	Wildlife Reserve, Al Maha	F	5	NO CHIP INFO	neg	neg. 3.1	X
NSw30	Drom1882	Wildlife Reserve, Al Maha	F	14	NO CHIP INFO	neg	pos. 160.9	X
NSw32	Drom1884	Wildlife Reserve, Al Maha	M	12	NO CHIP INFO	neg	pos. 101.3	X
NSw34	Drom1886	Wildlife Reserve, Al Maha	M	7	NO CHIP INFO	neg	neg. 18.3	X
NSw35	Drom1887	Wildlife Reserve, Al Maha	M	2 y 1 m	NO CHIP INFO	neg	neg. 3.1	X
NSw38	Drom1890	Wildlife Reserve, Alpha	M	15	NO CHIP INFO	neg	pos. 152.0	X
NSw40	Drom1892	Wildlife Reserve, Travco	M	12	NO CHIP INFO	neg	pos. 175.9	X
NSw42	Drom1893	Wildlife Reserve, Travco	M	9	NO CHIP INFO	neg	pos. 162.8	X
NSw43	Drom1894	Wildlife Reserve, ArabAdv	F	16	NO CHIP INFO	neg	pos. 121.5	X
NSw46	Drom1897	Wildlife Reserve, ArabAdv	M	16	NO CHIP INFO	neg	pos. 182.9	X
NSw48	Drom1899	Wildlife Reserve, ArabAdv	M	12	NO CHIP INFO	neg	pos. 147.0	X
NSw51	Drom1901	Wildlife Reserve, ArabAdv	F	16	NO CHIP INFO	neg	neg. 26.1	X
NSw52	Drom1902	Wildlife Reserve, ArabAdv	M	11	NO CHIP INFO	neg	pos. 95.0	X
NSw53	Drom1903	Wildlife Reserve, ArabAdv	F	2	NO CHIP INFO	neg	neg. 3.6	X
NSw54	Drom1904	Wildlife Reserve, ArabAdv	M	6	NO CHIP INFO	neg	pos. 40.8	X
NSw55	Drom1905	Wildlife Reserve, ArabAdv	F	18	NO CHIP INFO	neg	pos. 170.2	X
NSw56	Drom1906	Wildlife Reserve, ArabAdv	F	6 m	NO CHIP INFO	neg	pos. 86.5	X
NSw57	Drom1907	Wildlife Reserve, DesertStar	M	8-9	NO CHIP INFO	neg	pos. 152.2	X
NSw59	Drom1909	Wildlife Reserve, DesertStar	M	5	NO CHIP INFO	neg	neg. 3.4	X
NSw60	Drom1910	Wildlife Reserve, DesertStar	M	5	NO CHIP INFO	neg	pos. 133.8	X
NSw63	Drom1913	Wildlife Reserve, DesertStar	M	15	NO CHIP INFO	neg	pos. 172.0	X
w67	Drom1917	Wildlife Reserve, DesertStar	M	18	NO CHIP INFO	neg	pos. 122.2	X
w69	Drom1919	Wildlife Reserve, DesertStar	M	18	NO CHIP INFO	neg	pos. 250.4	X
w79	Drom1928	Wildlife Reserve, DesertStar	M	7	NO CHIP INFO	neg	pos. 224.2	X
M1	Drom1929	Bedouin farm, Al Mazrooei	F	4	NO CHIP INFO	neg	neg. 5.9	X
M2	Drom1930	Bedouin farm, Al Mazrooei	F	4	NO CHIP INFO	neg	neg. 5.5	X
M3	Drom1931	Bedouin farm, Al Mazrooei	F	4	NO CHIP INFO	neg	pos. 226.8	X
M4	Drom1932	Bedouin farm, Al Mazrooei	F	15	NO CHIP INFO	neg	pos. 235.1	X
M5	Drom1933	Bedouin farm, Al Mazrooei	F	8	NO CHIP INFO	neg	pos. 86.9	X
M6	Drom1934	Bedouin farm, Al Mazrooei	F	25-30	NO CHIP INFO	neg	pos. 152.9	X
M7	Drom1935	Bedouin farm, Al Mazrooei	F	5	NO CHIP INFO	neg	neg. 5.9	X

M8	Drom1936	Bedouin farm, Al Mazrooei	F	12	NO CHIP INFO	neg	pos. 70.3	X
M9	Drom1937	Bedouin farm, Al Mazrooei	F	12	NO CHIP INFO	neg	pos. 113.6	X
M10	Drom1938	Bedouin farm, Al Mazrooei	F	12	NO CHIP INFO	neg	pos. 244.1	X
M11	Drom1939	Bedouin farm, Al Mazrooei	F	14	NO CHIP INFO	neg	pos. 188.0	X
M12	Drom1940	Bedouin farm, Al Mazrooei	M	2 m	NO CHIP INFO	neg	pos. 120.8	X
M13	Drom1941	Bedouin farm, Al Mazrooei	M	10	NO CHIP INFO	neg	pos. 196.4	X
M14	Drom1942	Bedouin farm, Al Mazrooei	M	14	NO CHIP INFO	neg	pos. 167.1	X
M15	Drom1943	Bedouin farm, Al Mazrooei	F	10	NO CHIP INFO	neg	pos. 151.7	X

Table S2. List of 100 selected IR genes.

Gene ID	Genes-No.SNPs	Name	Description
**MHC Class I**			
Cadr_00022140	112	HLA-A-24	HLA class I histocompatibility antigen, A-24 alpha chain (Homo sapiens OX=9606)
Cadr_00022145	30	HLA-A-11	HLA class I histocompatibility antigen, A-11 alpha chain (Homo sapiens OX=9606)
Cadr_00022149	14	HLA-A-69	HLA class I histocompatibility antigen, A-69 alpha chain (Homo sapiens OX=9606)
Cadr_00022150	134	HLA-A-30	HLA class I histocompatibility antigen, A-30 alpha chain (Homo sapiens OX=9606)
Cadr_00022148	20	HLA-C	HLA class I histocompatibility antigen, Cw-6 alpha chain (Homo sapiens OX=9606)
Cadr_00022156	4	Patr	class I histocompatability B-1 alpha chain (Fragment) (Pan troglodytes OX=9598)
Cadr_00022105	18	Patr-A-126	Patr class I histocompatibility antigen, A-126 alpha chain (Pan troglodytes OX=9598)
Cadr_00022139	67	Patr-A-126	Patr class I histocompatibility antigen, A-126 alpha chain (Pan troglodytes OX=9598)
Cadr_00022147	5	Patr-A-126	Patr class I histocompatibility antigen, A-126 alpha chain (Pan troglodytes OX=9598)
Cadr_00022160	31	Patr-A-126	Patr class I histocompatibility antigen, A-126 alpha chain (Pan troglodytes OX=9598)
Cadr_00022155	0	Popy	class I histocompatability antigen A-1 alpha chain (Pongo pygmaeus OX=9600)
**MHC Class II**			
Cadr_00022027	28	BoLA-DQB	BoLa class II histocompatability antigen, DQB*0101 beta chain (Bos taurus OX=9913)
Cadr_00004894	11	CD74	HLA class II histocompatability antigen gamma chain (Homo sapiens OX=9606)
Cadr_00022030	95	DLA	class II histocompatability antigen, DR-1 beta chain (Canis lupus familiaris OX=9615)
Cadr_00022020	5	HLA-DMA	HLA class II histocompatability antigen, DM alpha chain (Homo sapiens OX=9606)
Cadr_00022021	33	HLA-DMB	HLA class II histocompatability antigen, DM alpha chain (Homo sapiens OX=9606)
Cadr_00022018	25	HLA-DOA	HLA class II histocompatability antigen, DO alpha chain (Homo sapiens OX=9606)
Cadr_00022026	72	HLA-DOB	HLA class II histocompatability antigen, DO alpha chain (Homo sapiens OX=9606)
Cadr_00022017	32	HLA-DPA1	HLA class II histocompatability antigen, DP alpha chain (Homo sapiens OX=9606)
Cadr_00022016	41	HLA-DPB1	HLA class II histocompatability antigen, DP alpha chain (Homo sapiens OX=9606)
Cadr_00022036	89	HLA-DRB1	HLA class II histocompatability antigen, DRB1-4 alpha chain (Homo sapiens OX=9606)
Cadr_00022037	63	HLA-DRB1	HLA class II histocompatability antigen, DRB1-1 alpha chain (Homo sapiens OX=9606)
Cadr_00022038	34	Mamu-DRA	Mamu class II histocompatibility antigen, DR alpha chain (Macac mulata OX=9544)
Cadr_00022032	7	RT1-Bb	Rano class II histocompatibility antigen, B-1 beta chain (Rattus norvegicus OX=10116)
Cadr_00022034	36	RT1-Bb	Rano class II histocompatibility antigen, B-1 beta chain (Rattus norvegicus OX=10116)
Cadr_00022028	15	SLA	class II histocompatibility antigem, DQ haplotype D alpha chain (Sus scrofa =X=9823)
Cadr_00022033	6	SLA	class II histocompatibility antigem, DQ haplotype D alpha chain (Sus scrofa =X=9823)
Cadr_00022035	4	SLA	class II histocompatibility antigem, DQ haplotype D alpha chain (Sus scrofa =X=9823)
**TLR**			
Cadr_00002152	5	TLR1	Toll-like receptor 1 (Homo sapiens OX=9606)
Cadr_00002153	23	TLR10	Toll-like receptor 10 (Bos taurus OX=9913)
Cadr_00001385	10	TLR2	Toll-like receptor 2 (Equus caballus OX=9796)
Cadr_00026583	31	TLR3	Toll-like receptor 3 (Boselaphus tragocamelus OX=9917)
Cadr_00016120	8	TLR4	Toll-like receptor 4 (Sus scrofa OX=9823)
Cadr_00023195	4	TLR5	Toll-like receptor 5 (Homo sapiens OX=9606)
Cadr_00002151	15	TLR6	Toll-like receptor 6 (Homo sapiens OX=9606)
Cadr_00003728	18	TLR7	Toll-like receptor 7 (Homo sapiens OX=9606)
Cadr_00003726	7	TLR8	Toll-like receptor 8 (Homo sapiens OX=9606)
Cadr_00020415	3	TLR9	Toll-like receptor 9 (Sus scrofa OX=9823)
**Granzyme**			
Cadr_00004168	4	GZMA	Granzyme A (Bos taurus OX=9913)
Cadr_00004169	1	GZMA	Granzyme A (Homo sapiens OX=9606)
Cadr_00005822	0	GZMB	Granzyme B (Homo sapiens OX=9606)
Cadr_00005823	22	GZMB	Granzyme B (Homo sapiens OX=9606)
Cadr_00005821	5	GZMH	Granzyme H (Homo sapiens OX=9606)
Cadr_00004167	6	GZMK	Granzyme K (Homo sapiens OX=9606)
Cadr_00025032	16	GZMM	Granzyme M (Homo sapiens OX=9606)
**Interleukin**			
Cadr_00001885	3	CXCL8	Interleukin-8 (Canis lupus familiaris OX=9615)
Cadr_00023412	7	IL10	Interleukin-10 (Lama glama OX=9844)
Cadr_00028914	14	IL10RA	Interleukin-10 receptor subunit alpha (Homo sapiens OX=9606)
Cadr_00001098	61	IL10RB	Interleukin-10 receptor subunit alpha (Homo sapiens OX=9606)
Cadr_00029940	16	IL1A	Interleukin-1 alpha (Lama glama OX=9844)
Cadr_00029941	20	IL1B	Interleukin-1 beta (Lama glama OX=9844)
**Killer cell**			
Cadr_00029273	19	Klra2	Killer cell lectin-like receptor 2 (Mus musculus OX=10090)
Cadr_00029303	4	KLRB1	Killer cell lectin-like receptor subfamily B member 1 (Homo sapiens OX=9606)
Cadr_00029300	2	Klrb1b	Killer cell lectin-like receptor subfamily B member 1B allele A (Camelus bactrianus XP_010944886.1)
Cadr_00029489	18	KLRC2	NKG2-C type II integral membrane protein (Homo sapiens OX= 9606)
Cadr_00029281	10	KLRD1	Natural killer cells antigen CD94 (Bos taurus OX=9913)
Cadr_00029283	13	Klre1	Killer cell lectin-like receptor subfamily E member 1 (Mus musculus OX=10090)

Cadr_00029297	73	KLRF1	Killer cell lectin-like receptor subfamily F member 1 (Macaca fascicularis OX=9541)
Cadr_00029295	48	KLRF2	Killer cell lectin-like receptor subfamily F member 2 (Homo sapiens OX=9606)
Cadr_00008447	42	Klrg2	Killer cell lectin-like receptor subfamily G member 2 (Mus musculus OX=10090)
Cadr_00029277	28	Klri1	Killer cell lectin-like receptor subfamily I member 1 (Mus musculus OX=10090)
Cadr_00029276	11	KLRK1	NKG2-D type II integral membrane protein (Pongo Pygmaeus OX=9600)
Cadr_00029279	14	KLRK1	NKG2-D type II integral membrane protein (Sus scrofa OX=9823)
**Other genes**			
Cadr_00030052	11	ACO1	Cytoplasmic aconitate hidratase (Bos taurus OX=9913)
Cadr_00020478	5	APPL1	DCC-interacting protein 13-alpha (Homo sapiens OX=9606)
Cadr_00002239	297	CC2D2A	Coiled-coil and C2 domain-containing protein 2A (Homo sapiens OX=9606)
Cadr_00029296	27	CLEC2B	C-type lectin domain family 2 member B (Homo sapiens OX=9606)
Cadr_00007342	11	CXCR2	C-X-C chemokine receptor type 2 (Bos taurus OX=9913)
Cadr_00030053	90	DDX58	Probable ATP-dependent RNA helicase DDX58 (Sus scrofa OX=9823)
Cadr_00020479	129	DNAH7	Dynein heavy chain 7 axonemal (Homo sapiens OX=9607)
Cadr_00006877	64	DPP4	Dipeptidyl peptidase 4 (Bos taurus OX=9913)
Cadr_00012213	12	FCAR	Immunoglobulin alpha Fc receptor (Homo sapiens OX=9606)
Cadr_00024638	47	FCRL3	Fc receptor-like protein 3 (Homo sapiens OX=9606)
Cadr_00011189	2	HP	Haptoglobin (Sus scrofa OX=9823)
Cadr_00006880	29	IFIH1	Interferon-induced helicase C domain-containing protein 1 (Homo sapiens OX=9606)
Cadr_00015578	0	IFNB2	Interferon beta-2 (Bos taurus OX=9913)
Cadr_00017035	3	IFNG	Interferon gamma (Camelus bactrianus OX=9837)
Cadr_00001103	50	IFNGR2	Interferon gamma receptor 2 (Homo sapiens OX=9606)
Cadr_00029272	12	MAGOHB	Protein mago nashi homolog 2 (Bos taurus OX=9913)
Cadr_00004186	13	MAP3K1	Mitogen-activated protein kinase kinase kinase 1 (Homo sapiens OX=9606)
Cadr_00005819	1	Mast	cell protease 3 (Ovis aries OX=9940)
Cadr_00012215	15	NCR1	Natural cytoxicity triggering receptor 1 (Bos taurus OX=9913)
Cadr_00021869	9	NCR2	Natural cytoxicity triggering receptor 2 (Homo sapiens OX=9606)
Cadr_00001692	77	NFKB1	Nuclear factor NF-kappa-B p105 subunit (Canis lupus familiaris OX=9615)
Cadr_00009474	6	NFKB2	Nuclear factor NF-kappa-B p100 subunit (Homo sapiens OX=9606)
Cadr_00029278	15	NKG2A	NKG2-A/NKG2-B type II integral membrane protein (Macaca mulatta OX=9544)
Cadr_00029993	12	NKL	Antimicrobial peptide NK-lysin (Fragment) (Sus scrofa OX=9823)
Cadr_00009139	6	PRF1	Perforin-1 (Homo sapiens OX=9606)
Cadr_00007027	22	PRKRA	Interferon-inducible double-stranded RNA_dependent protein kinase activator A (Homo sapiens OX=9606)
Cadr_00009475	0	Psd	PH and SEC7 domain-containing protein 1 (Mus musculus OX=10090)
Cadr_00024639	2	PTMA	Prothymosin alpha (Pongo abelii OX=9601)
Cadr_00006681	163	PTPN4	Tyrosine-protein phosphatase non-receptor type 4 (Homo sapiens OX=9606)
Cadr_00001384	10	RNF175	RING finger protein 175 (Homo sapiens OX=9606)
Cadr_00004895	4	Rps14	40S ribosomal protein S14 (Mus nusculus OX=10090)
Cadr_00017710	6	Rps7	40S ribosomal protein S7 (Rattus norvegicus OX=10116)
Cadr_00001327	32	Suclg1	Succinate--CoA ligase [ADP/GDP-forming] subunit alpha, mitochondrial (Mus musculus OX=10090)
Cadr_00002785	18	Tmem255a	Transmembrane protein 255A (Mus musculus OX=10090)
Cadr_00022101	7	TNF	Tumor necrosis factor (Camelus bactrianus OX=9837
Cadr_00006503	45	Traf3	TNF receptor-associated factor 3 (Mus musculus OX=10090)
Cadr_00011190	1	TXNL4B	Thioredoxin-like protein 4B (Homo sapiens OX=9606)

Table S3. Potential splice sites in the *FCAR* and *CLEC2B* gene identified with NatGene2 v.2.42. Positions follow the CamDro3 reference genome (GCF_000803125.2**)**. Cut-off values used for confidence: Highly confident donor sites (H): 95.0%. The closest splice sites to significant SNPs (Table 1; Figure S2) are highlighted in bold.

***FCAR* (chr9: 73807328 - 73825701)**

Donor splice sites, direct strand (pos 5'->3')	phase	strand	confidence	5' exon^intron 3'
73807601	1	+	0.88	TCCAACACCG^GTGAGTACAG
73809007	1	+	0.45	CAAGATGAAC^GTAGGGAGAC
73809252	0	+	0.34	GTGGGTGGAC^GTAAGATGTG
73809567	1	+	0.44	TGAAGACGAG^GTCAGACCAG
73809735	0	+	0.47	CACCTGGGAG^GTGAATACCA
73809757	1	+	0.55	ATTCTTCTGA^GTGAGTTCAG
73810248	1	+	0.94	GCCAAAAAGA^GTAAGTCTCA H
73810301	0	+	0.31	GGGAGGTGGG^GTGAGGGTGC
73810315	0	+	0.41	GGGTGCGCAG^GTGTGTGTCC
73810569	0	+	0.34	CTTCAAAGAG^GTAAAATTTC
73810756	0	+	0.77	GAACAGGGCG^GTAGGTTCTC
73811382	1	+	0.46	TTCTATAGAG^GTACGGCGTC
73812004	0	+	0.44	TCCATCTTCA^GTGAGTGAGC
73812707	1	+	0.65	TATCATTCAG^GTAATTTCAC
73813597	2	+	0.45	AGAGACACCT^GTAAGCCCTG
73813900	0	+	0.92	AGCATCGCTG^GTGAGCAGCA H
73814220	0	+	0.47	TGGGGACAAG^GTGAGACTCT
73814382	0	+	0.3	CAACCAGCGC^GTGAGGCAGT
73814631	0	+	0.83	GAAGCTAGAG^GTAAGACAAG
73814706	0	+	0.34	ATATGACAAG^GTACTTTCTT
73815102	1	+	0.82	GTTGCCCACT^GTGAGTCATT
73815812	0	+	0.42	TATCTCTGAG^GTCAGTGGGT
73815987	1	+	0.63	ACTGCTCACG^GTGAGTGGAT
73817375	2	+	0.32	ATATATGAAG^GTTGGTGAAA
73817612	0	+	0.49	GTACAGCATG^GTGAGTAGAA
73818627	1	+	0.93	TTCTGCCTTG^GTGAGTCCTG H
73819125	1	+	0.81	GCACAGGATG^GTAAGCATTC
73820437	0	+	0.34	GGAAGGAACT^GTAAGTCCTT
**73820819**	**1**	**+**	**0.91**	**GTGGTGACAG^GTGCGGAGGC H**
73821287	2	+	0.56	TCAACCACTC^GTGAGTTTAT
73821306	0	+	0.47	TGTTAATGTG^GTGAGTTTTG

73822423	1	+	1	GTGGTCACAG^GTAAGGGCAC H
73823183	1	+	0.56	CAGTGCCAAG^GTAATCTGCT
73824511	0	+	0.41	GTTCCGTGAC^GTAAGTCTGC
73825185	0	+	0.34	GAACACACCT^GTGAGTGACA
73825399	1	+	0.46	ACACTGAATA^GTAAGTACAT
73825617	1	+	0.00	CTCAGAAATG^GTATGATTTG

***CLEC2B* (chr34: 15807876 - 15823606)**

Donor splice sites, direct strand (pos 5'->3')	phase	strand	confidence	5' exon^intron 3'
15808104	0	+	0.31	GGAACCCAGG^GTACCAGGGG
**15808984**	**1**	**+**	**0.99**	**AAGAAGGAAG^GTAAGAACAT H**
15809097	0	+	0.37	TTCTACCAAG^GTGGGGGGGG
15809635	1	+	0.47	TTCAGAGTAG^GTAAGATCCA
15809711	0	+	0.37	TCAAAATAGG^GTGAGGCAGG
15809776	1	+	0.44	GGGGGCTATG^GTGAGTTTTA
15809915	0	+	0.35	TCTGTCTAAG^GTTAGATCAA
15812452	1	+	0.32	TATGTCAAAG^GTGGGGTGGA
15813041	1	+	0.37	AAAAAAAAAG^GTGACTTGTC
15813559	1	+	0.34	AAGGACACAT^GTAAGAAGTA
15813634	1	+	0.54	GTTCTCCAAG^GTGAGTACAT
15813936	1	+	0.47	ATTGCCAAAG^GTTAGTTTTG
15814738	2	+	0.41	TGATGACTGA^GTGAGTGTGT
15816556	0	+	1	AGAAAAGATG^GTAAGTAATT H
15816990	2	+	0.46	ATGTGTCCCT^GTAAGTTTCC
15817564	1	+	0.32	GAAAAACTAG^GTATAGAGCA
15819286	0	+	0.7	GGCTCAGAGG^GTAAAATGCT
15819547	1	+	0.81	TTAAGAATTG^GTGAGTTTCT
15820366	0	+	0.36	GTAACTTTAT^GTAAGTTTAA
15820700	1	+	0.7	AAAACTATGA^GTAAGATGGT
15822616	1	+	0.37	GGAAGAAAGG^GTGTGTGGGC

Table S4. **Linkage Disequilibrium-based haplotype (gene-set) test showing 40 genes with significant SNPs at p < 0.05.** Identified candidate gene FCAR was nominally significant (p < 0.01) indicated with *. NSNP - Number of SNPs in set; NSIG - Total number of SNPs below p-value threshold; ISIG - Number of significant SNPs also passing LD-criterion; STAT - Average test statistic based on ISIG SNPs; EMP1 - Empirical set-based p-value; SNPs - positions of SNPs in the set.

SET	NSNP	NSIG	ISIG	EMP1	SNPs	Name
**Cadr_00012213**	**12**	**9**	**1**	**0.0031***	**9_73819886**	**FCAR**
Cadr_00006880	29	17	1	0.0126	5_36852218	IFIH1
Cadr_00029278	15	12	1	0.01365	34_15453059	NKG2A
Cadr_00025032	15	1	1	0.0197	22_299371	GZMM
Cadr_00022016	41	9	1	0.0221	20_20681619	HLA-DPB1
Cadr_00006681	162	2	2	0.0254	5_8524970|5_8553067	PTPN4
Cadr_00017035	3	1	1	0.0281	12_24456808	IFNG
Cadr_00001327	32	1	1	0.0486	2_5563103	Suclg1
Cadr_00029276	11	1	1	0.05214	34_15423950	KLRK1
Cadr_00029277	28	17	3	0.05869	34_15447536|34_15432256|34_15441877	Klri1
Cadr_00022020	5	1	1	0.06124	20_20736642	HLA-DMA
Cadr_00009474	6	1	1	0.07274	11_60541518	NFKB2
Cadr_00022160	25	2	1	0.08034	20_23402080	Patr-A-126
Cadr_00022033	2	2	1	0.08489	20_20924560	SLA-DQS
Cadr_00022017	31	2	2	0.09119	20_20688614|20_20690464	HLA-DPA1
Cadr_00022030	95	2	1	0.09369	20_20871804	DLA-DR1
Cadr_00029273	19	5	3	0.1026	34_15379486|34_15372199|34_15371765	Klra2
Cadr_00022018	25	4	2	0.1056	20_20701339|20_20701643	HLA-DOA
Cadr_00007027	22	4	1	0.1096	5_49754009	PRKRA
Cadr_00028914	14	7	2	0.1155	33_12221507|33_12212516	IL10RA
Cadr_00029297	73	4	1	0.1157	34_15833105	KLRF1
Cadr_00022021	33	5	2	0.142	20_20746290|20_20745925	HLA-DMB
Cadr_00029489	18	10	1	0.1641	34_22473182	KLRC2
Cadr_00001103	49	9	2	0.184	1_113858111|1_113864038	IFNGR2
Cadr_00030053	89	3	3	0.2045	Contig45_329479|Contig45_327196|Contig45_324930	DDX58
Cadr_00004894	11	1	1	0.2159	3_109719975	CD74
Cadr_00022139	67	9	1	0.2391	20_23039666	Patr-A-126
Cadr_00022026	72	8	1	0.2518	20_20828643	HLA-DOB
Cadr_00022150	128	12	2	0.2689	20_23268542|20_23270977	HLA-A-30
Cadr_00022027	28	1	1	0.2763	20_20838772	BoLA-DQB
Cadr_00022148	20	3	1	0.2962	20_23247823	HLA-Cw6
Cadr_00022038	34	1	1	0.3012	20_21060760	Mamu-DRA
Cadr_00029272	12	1	1	0.3017	34_15363470	MAGOHB
Cadr_00020479	127	2	1	0.3147	17_23843066	DNAH7
Cadr_00002153	23	3	2	0.3245	2_95082961|2_95084365	TLR10
Cadr_00006877	64	6	1	0.3928	5_36650034	DPP4
Cadr_00022149	14	2	1	0.4345	20_23254824	HLA-A-69
Cadr_00024638	47	4	2	0.4364	21_21451949|21_21438740	FCRL3

Cadr_00001692	76	2	2	0.4563	2_49863272|2_49851559	NFKB1
Cadr_00022140	110	3	2	0.6527	20_23101931|20_23103971	HLA-A-24
